# Factors Affecting Cryopreservation of Domestic Cat (*Felis catus*) Epididymal Spermatozoa

**DOI:** 10.3390/ani15070949

**Published:** 2025-03-26

**Authors:** Natalia Gañán, Ana Sanchez-Rodriguez, Eduardo R. S. Roldan

**Affiliations:** Department of Biodiversity and Evolutionary Biology, Museo Nacional de Ciencias Naturales (CSIC), 28006 Madrid, Spain; natalia.gannan@gmail.com (N.G.); anasanchez@mncn.csic.es (A.S.-R.)

**Keywords:** epididymis, sperm, cryopreservation, freezing, refrigeration, domestic cat

## Abstract

Sperm cryopreservation is a powerful tool for the conservation of endangered species, but its application requires adapting protocols to particular species, due to differences in sperm structure, function, and cryosensitivity. Research on the biology of endangered felids primarily relies on the domestic cat as an experimental model. Semen from live animals can be collected using several methods. However, in animals that die due to roadkill or in the field, spermatozoa must be retrieved from the epididymis. Differences may exist in the cryosensitivity of epididymal and ejaculated sperm due to the influence of secretions from accessory genital glands. We analyzed the effects of several factors on the motility and acrosomal integrity of cryopreserved cat epididymal spermatozoa, including cooling rate, storage system, time and temperature of straw loading, and the freezing method in nitrogen vapors. There were no significant differences in motility or acrosomal integrity at thawing between fast (−0.5 °C/min) or slow (−0.125 °C/min) cooling rates or between loading straws at room temperature versus 5 °C. Post-thaw motility was significantly higher when using straws compared to pellets and when freezing in nitrogen vapors at two levels rather than at a single level. Additionally, interactions between the loading temperature of straws and both motility and acrosomal integrity were not significant. These results are important for standardizing protocols to cryopreserve feline epididymal sperm, facilitating the rescue of genetic material from endangered species in the field.

## Simple Summary

The rescue of genetic material and gametes is an important tool in conservation efforts. When animals die in the field, due to roadkill or traps, after reintroduction efforts, or when dispersing to new habitats, it is crucial to recover gametes for storage in genome resource banks. One possible method to rescue spermatozoa is by collecting them from the epididymis and processing them for cryopreservation. Several factors may influence the success of sperm retrieval, refrigeration, and freezing under optimal conditions. These factors affect the subsequent viability, motility, and structural integrity of spermatozoa upon thawing, as well as their potential use in assisted reproduction. This study examined the effects of cooling rate (slow vs. fast), packaging system (straws vs. pellets), the temperature at which straws are loaded, and freezing method (single- or two-level exposure to nitrogen vapors). Assessments of motility and acrosome integrity showed minimal differences due to cooling temperature but demonstrated a clear advantage when using straws over pellets and when freezing in a two-level system. These findings are important for the future rescue of genetic material from feline species in the wild.

## 1. Introduction

The importance of sperm cryopreservation as a tool for the conservation of endangered species has been widely justified [1,2,3,4,5]. Developing cryopreservation protocols requires assessing how various factors involved in freezing and subsequent thawing affect spermatozoa of a given species. In endangered animals, these assessments are particularly challenging due to the limited availability of samples. Therefore, experimental models are used in such cases. These models are more readily available and phylogenetically close to the endangered species of interest. This allows work with a sufficient number of samples and to perform tests needed to refine techniques before adapting them to the wild species of interest [6,7,8]. For studies on the sperm biology of wild felids, the domestic cat (*Felis catus*) serves as the primary experimental model [6,9,10,11,12,13].

Several studies have analyzed the effects of factors involved in the cryopreservation of cat spermatozoa on different sperm parameters. The goal has been to optimize a sperm preservation protocol that ensures high fertilization rates, particularly in in vitro fertilization [14,15,16,17,18,19], intracytoplasmic sperm injection [20], or artificial insemination [13,21,22,23,24,25]. Key factors in cryopreservation include the semen extenders used [26,27,28] and the type of cryoprotectants employed [29]. Also important are the freezing and thawing speeds, as spermatozoa of different species exhibit varying degrees of cryosensitivity to thermal shock caused by low temperatures [2,30,31,32]. Thus, the effect of cooling [33,34], freezing [35], and thawing [24] rates were found to impact on cat sperm motility, viability, structural integrity, or functionality upon thawing. Additionally, the size and composition of the storage vessel (e.g., ampoules, pellets, straws, or cryovials) play a crucial role, as temperature variation depends on the thermal conductivity of the container [33,36].

Spermatozoa can be obtained through various methods, namely, following ejaculation into an artificial vagina or by means of electrical stimulation, collecting them using urethral catheterization, or retrieval from the epididymis following castration or death [37,38]. The main difference between spermatozoa collected through these methods is their exposure to fluids of the accessory glands constituting the seminal plasma. Proteins and lipids in seminal plasma may coat sperm and contribute to functional roles, such as preventing premature capacitation or facilitating interactions with the female reproductive tract.

In certain circumstances, particularly in endangered species, only epididymal spermatozoa can be recovered. Therefore, it is crucial to determine whether differences exist between epididymal and ejaculated sperm, and whether epididymal sperm can be effectively cryopreserved and used for assisted reproduction [37]. No significant differences seem to exist in motility or acrosomal integrity between cryopreserved ejaculated and epididymal cat sperm when the same freezing protocol is used for both [39]. Electroejaculated and epididymal cat sperm are not highly susceptible to cold shock when refrigerated at moderate cooling rates to 4 °C showing reasonable survival rates after freezing and thawing [40]. In addition, the chromatin structure of both ejaculated and epididymal cat sperm shows good tolerance to cold storage and cryopreservation [41].

However, pregnancy rates achieved with epididymal sperm are lower than those obtained with ejaculated sperm. This trend is observed in both vaginal non-surgical insemination [42] and surgical insemination directly into the oviduct [43]. The lower success rate may be further exacerbated when using cryopreserved sperm, as post-thaw survival is reduced and sperm interactions with the female tract may be compromised after insemination [42].

The objective of this study was to evaluate factors and conditions involved in the cryopreservation process of cat epididymal spermatozoa, serving as a model for endangered felids. The experiments examined the efficacy of cryopreservation by analyzing sperm motility and acrosomal integrity before and after freezing using the following conditions: (a) two cooling rates: fast cooling for 30 min (−0.5 °C/min) and slow cooling for 120 min (−0.125 °C/min), (b) two storage systems: pellets on dry ice (solid CO_2_) and straws on nitrogen vapors, (c) two straw filling methods: at room temperature before cooling and at 5 °C after cooling, and (d) two straw freezing methods, with two freezing rates: in nitrogen vapors, either at a constant or at varying distances from the liquid nitrogen surface, in a one- or two-level system, respectively.

## 2. Materials and Methods

### 2.1. Animals

The testes and epididymides used for this study came from male domestic cats (a total of 134 males, 1.54 ± 0.13 years old) orchiectomized in veterinary clinics and animal protection societies of the municipality of Madrid. Immediately after castration, testes and epididymides were placed in a self-sealing plastic bags (11 × 7 cm) and transported to the laboratory inside an isothermal container (25 × 17 × 11.5 cm) at room temperature (~20 °C). Once in the laboratory, epididymides were separated from the testes and were kept at this temperature until processing, which took place between 3–12 h post-surgery.

### 2.2. Epididymal Sperm Collection

The spermatozoa were retrieved from the epididymis and vas deferens by piercing the cauda epididymis in 0.4 mL of cryodiluent in a Petri dish (35 × 10 mm). The dish was kept at room temperature for 10 min to allow the exit of spermatozoa. The diluent used was TEST, composed of 4.83% Tes, 1.15% Tris, 0.4% glucose, 20% egg yolk, 200 i.u/mL penicillin, 200 µg/mL streptomycin, and a final glycerol concentration of 4% (pH = 7.2 and osmolality = 360 mOsmol/kg). Reagents used to prepare cryodiluents and other solutions were from Sigma (Madrid, Spain), unless stated otherwise.

Samples were taken for assessments of motility, sperm concentration, morphological abnormalities, and acrosome integrity (see below). The rest of the sperm suspension was carefully transferred from the Petri dish to a 1.5 mL polystyrene microtube, measuring and recording the volume in order to subsequently calculate the total number of spermatozoa (and motile spermatozoa) recovered. Samples that did not have a minimum of 40% motile sperm and a concentration of 20 × 10^6^ sperm/mL were discarded from the experiments. Concentration was adjusted with addition of diluent to result in final concentration of 5–10 × 10^6^ spermatozoa per straw or pellet.

### 2.3. Sperm Cryopreservation

Samples were randomly assigned to different experimental groups to examine various aspects of the cryopreservation process: refrigeration, packaging system, temperature and timing of straw loading, and freezing.

#### 2.3.1. Refrigeration

**Figure 1:**
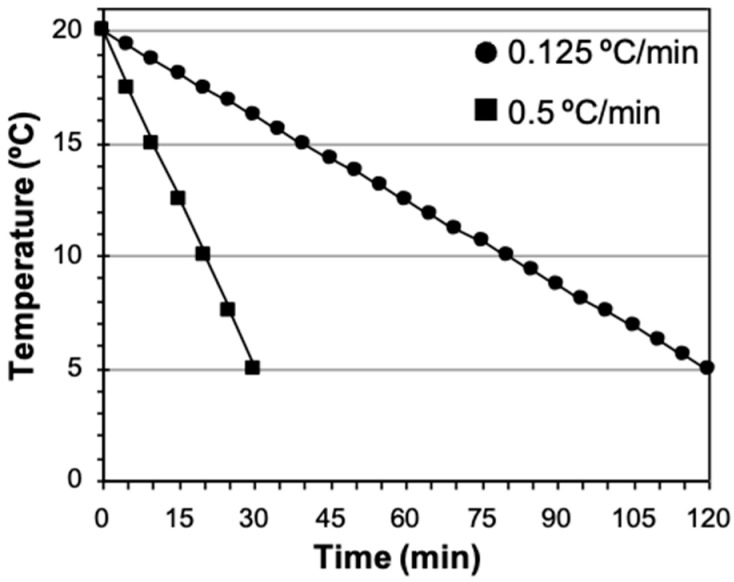
Cooling curves used with domestic cat spermatozoa: fast, cooling samples over 30 min (−0.5 °C/min) and slow, cooling samples over 120 min (−0.125 °C/min).

Two cooling rates were used to refrigerate sperm in diluent (*n* = 21). (a) Fast rate, cooling sperm from 20 °C to 5 °C for 30 min (−0.5 °C/min). This cooling rate was achieved placing a microtube containing the sperm suspension in a glass beaker with 150 mL of milli-Q water which, in turn, was placed in a freezer (−20 °C) for 30 min. During this period of time the temperature of samples went down from 20 °C to 5 °C (Figure 1). (b) Slow rate, bringing the sperm suspension from 20 °C to 5 °C over a period of 120 min (−0.125 °C/min). The sperm suspension (in a microtube or already loaded in straws) was placed on a floating rack inside an isothermal container with 1 L of water at room temperature (20 °C) and decreasing the water temperature by adding ice, dry ice, or cold water at preset times until a temperature of 5 °C was reached (Figure 1).

After refrigeration using the fast or the slow cooling rates, an aliquot kept in a microtube and refrigerated under the same conditions as the sample to be frozen was used to evaluate motility and acrosome integrity.

The fast and slow cooling rates and the conditions necessary to achieve a descent in temperature in the period of interest (30 or 120 min) were defined in earlier studies in our laboratory. Temperatures were monitored with a digital thermometer.

#### 2.3.2. Packaging System

After refrigeration, the sperm samples were frozen in pellets on dry ice (solid CO_2_ at −79 °C) or in straws on nitrogen vapors, as appropriate. Samples (*n* = 19) were randomly assigned to the different treatments. When using pellets, a block of dry ice was previously prepared in which small perforations were made with a mold built using a block of wood and nails whose heads had been removed. A total of 30 µL of sperm suspension was placed in each well using an automatic pipette fitted with a yellow tip. When using straws, 0.25 mL straws (Minitübe, Tiefenbach, Germany) were used, cut in half with scissors, and discarding the open end without the cotton plug. For each straw, 50 µL of sperm suspension were loaded aspirating with an automatic pipette fitted with a tip. Air was then aspirated by turning the pipette plunger to move the sperm column towards the center of the straw. After filling, each straw was closed with a heat sealer (ERSA, Minitübe) and forceps.

#### 2.3.3. Temperature and Timing of Straw Loading

Two different conditions were assessed (*n* = 10 each): (a) Filling straws at room temperature, prior to refrigeration. After taking subsamples for evaluation of sperm parameters before refrigeration, the straws were loaded, sealed and placed in a large cryovial (5 mL; 7.5 cm in length) that was placed on a floating Styrofoam rack in a bath at room temperature for refrigeration. Inside the cryovial, the probe of a digital thermometer was inserted to monitor actual temperature drop of the samples (which varies slightly with that of the water bath). (b) Filling straws at 5 °C after refrigeration. After taking subsamples for the assessment of parameters before refrigeration, the sperm suspension was kept in a microtube and placed in a floating rack in a bath at room temperature for cooling. The probe of a digital thermometer was placed on the rack holding the microtube to monitor the temperature decrease. Once the temperature of the sperm suspension reached 5 °C, the straws were loaded in a cold chamber at the same temperature.

#### 2.3.4. Freezing

Two storage vessels were compared: pellets vs. straws. When freezing in pellets, the sperm suspension was deposited in the orifices on dry ice and allowed to freeze for 3 min; after that time the pellets were thrown into liquid nitrogen [44] and stored in labeled cryovials (2 mL; 3.5 cm long) perforated to allow entry of liquid nitrogen. The cryovials were placed in labeled rods that were stored in a liquid nitrogen tank.

**Figure 2:**
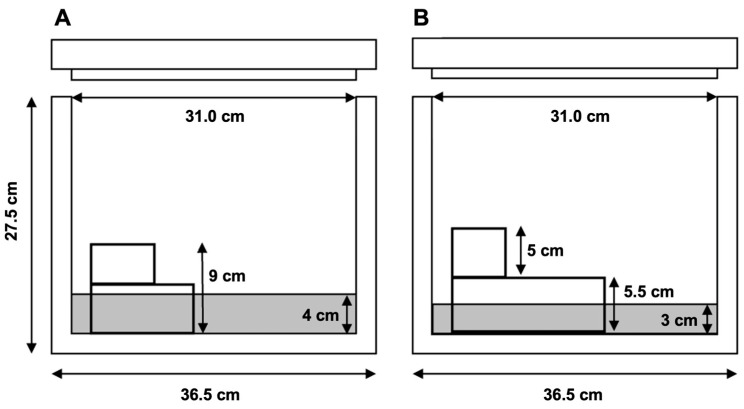
Diagrams of containers used for freezing straws over nitrogen vapors: (**A**) Freezing at one level, 5 cm from liquid nitrogen surface; (**B**) freezing using two levels, 7.5 and 2.5 cm from liquid nitrogen surface.

Straws were frozen over nitrogen vapors. Two methods to freeze straws were examined (*n* = 15) (Figure 2: a one-level and a two-level system. For the one-level system, liquid nitrogen was poured into an expanded polystyrene foam box to a depth of 4 cm (Figure 2). A metal rack was placed in the box, with a height that allowed the straws to be frozen in nitrogen vapors, 5 cm above the liquid nitrogen surface. Before freezing the straws, the box was covered for 5 min to equilibrate the temperature of the vapors inside the box. The straws were placed on the rack and left for 10 min with the lid closed. After the freezing time had elapsed, the straws were dipped into liquid nitrogen and stored in labeled cryovials (5 mL; 7.5 cm long), which were held in labeled rods, for storage in a liquid nitrogen tank. For the two-level system (Figure 2), the same expanded polystyrene box was used, but the liquid nitrogen was filled to a depth of 3 cm. A rack system was used to freeze the straws in the nitrogen vapors, first at 7.5 cm, and then at 2.5 cm above the liquid nitrogen level. For freezing, the straws remained in the upper level (7.5 cm) for 1 min and after this time they were transferred, in the same order in which they had been placed in the first level, and with a pre-cooled forceps, to the second level (2.5 cm) where they were left for a further 1 min [3]. Once frozen, the straws were dipped into liquid nitrogen and stored in labeled cryovials (5 mL; 7.5 cm long), which were placed in labeled rods, for storage in a liquid nitrogen tank.

### 2.4. Sperm Analysis After Thawing

As thawing medium, a Ham’s F-10 medium (Irvine Scientific, Izasa, Barcelona, Spain) was used, which was supplemented with inactivated fetal bovine serum (5%), L-glutamine (0.292 ng/mL), pyruvate (0.110 mg/mL), and antibiotics (130 i.u. penicillin/mL, 130 µg streptomycin/mL, and 260 µg neomycin/mL). For sample thawing, a pan with liquid nitrogen, a water bath at 37 °C and a thermoblock at 37 °C with sterile microtubes (1.5 mL) were used. The cryovial containing frozen pellets or straws was transferred from the liquid nitrogen tank where it was stored to the pan with liquid nitrogen and the pellets or straws to be thawed were extracted.

For the pellets, a glass tube (12 × 75 mm) with 120 µL of thawing medium was used. The tubes were placed in a 37 °C water bath. The pellet was removed from the nitrogen bath with pre-cooled forceps and quickly transferred to one of the tubes containing pre-warmed thawing medium, which was kept for 30 s in the bath, shaking it very gently to promote homogenization of the suspension. The sperm suspension was then transferred very slowly, with a pipette, from the glass tube to a pre-warmed microtube which was kept closed in a thermoblock at 37 °C.

The straws were thawed in a beaker containing sterile physiological solution (NaCl 0.9%, *w*/*v*) placed in a water bath at 37 °C. After removing the straw from liquid nitrogen, it was held with forceps by the end with the cotton plug for 10 s, then placed in the beaker, without releasing it from the forceps, for 30 s, shaking it carefully. After this time the straw was removed from the bath and dried with paper. The end sealed using heat was cut off, the straw placed vertically, with the open end near the bottom of a prewarmed microtube and the other end cut off so that the contents of the straw fell inside the vial. Once the sperm suspension was in the microtube, 150 µL of Ham’s F-10 medium with serum, glutamine, pyruvate, and antibiotics (composition given above) was added very slowly, drop by drop, tapping the tube gently to help homogenize the sperm suspension. A volume of 5 µL of diluted sperm suspension were taken for motility evaluation, whereas 10 µL of suspension were fixed with 4% paraformaldehyde for subsequent staining and evaluation of acrosomal integrity. The remaining sample was incubated for up to 270 min at 37 °C in a thermoblock under air. Aliquots were taken for evaluation of motility and acrosome integrity at hourly intervals, starting 90 min after thawing (Table 1).

### 2.5. Sperm Evaluation

The sperm parameters analyzed were motility, sperm concentration, acrosome integrity, and abnormal sperm.

For motility evaluation, a 5 µL aliquot of sperm suspension was taken and placed between a slide and a coverslip that were prewarmed on a plate at 37 °C. The sample was examined microscopically, on a warm stage at 37 °C, using phase contrast optics to assess motility. The percentage of motile spermatozoa and the quality of motility were estimated. The quality evaluation was made by an experienced observer using a scale from 0 (no movement) to 5 (progressive and fast movement) [44]. Motility and quality values were used to calculate a Sperm Motility Index (SMI) as follows: [(% motile + quality × 20)/2] [45].

To estimate sperm concentration, a volume of 5 µL of sperm suspension was fixed in 45 µL of 1% glutaraldehyde in 0.33 M cacodylate buffer (pH 7.3). A total of 10 µL of the suspension fixed in glutaraldehyde–cacodylate were placed in a Neubauer chamber (hemocytometer). At least two large opposite quadrants of the chamber were counted and the concentration was calculated from the average value obtained with the count and the dilution factor used.

To evaluate morphological abnormalities and acrosome integrity, 10 µL of sperm suspension were fixed in 250 µL of 4% paraformaldehyde [2 g of paraformaldehyde, 7.81 g Na_2_HPO_4_ anhydrous (110 mM), 0.172 g NaH_2_PO_4_·H_2_O (2.5 mM), in 500 mL of milli-Q water] in a polystyrene microtube (0.5 mL). The fixed samples were kept in the refrigerator (4 °C) until processing as previously described [46]. The fixed spermatozoa were centrifuged at 1700× *g* for 8 min, the supernatant was removed, and the pellet was resuspended in 250 µL of 0.1 M ammonium acetate (pH 9.0). The sample was re-centrifuged at the same speed and resuspended in 250 µL of ammonium acetate. After the second wash, the pellet was centrifuged for the last time at the same speed and approximately 200 µL of the supernatant was removed. The pellet was resuspended in the remaining supernatant by tapping the vial with the fingers (i.e., without pipetting). The sperm suspension was divided into two drops which were spread on two slides using a coverslip (22 × 22 mm) and allowed to air dry. After drying, the smears were stained with 40 µL of a Coomassie blue solution (0.22 g of dye in a solution composed of 50 mL of methanol, 10 mL of glacial acetic acid, and 40 mL of milli-Q water) for 90 s, washed with milli-Q water and allowed to air dry. Once the smears were dry, they were mounted with a few drops of DPX and a coverslip (40 × 60 mm) and left to dry protected from light until evaluation.

**Figure 3:**
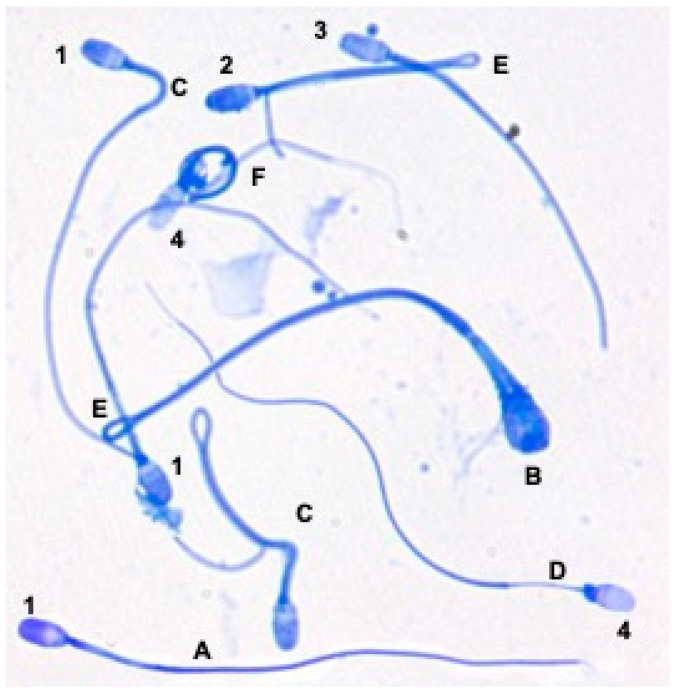
Acrosome integrity and morphological abnormalities in domestic cat spermatozoa evaluated using Coomassie blue-stained slides. Acrosome: (1) intact normal, (2) intact abnormal, (3) damaged and (4) missing. Morphology: (A) normal, (B) macrocephalic, (C) bent midpiece, (D) aplasic midpiece, (E) bent principal piece, (F) coiled tail.

For the evaluation of acrosome integrity, Coomassie blue-stained spermatozoa were assigned to four categories: (a) normal intact, those with the acrosomal zone uniformly stained blue; (b) abnormal intact, those in which some areas of the acrosome are more intensely stained blue than the rest; (c) damaged, those in which vesicles, bulges or partial detachment of the membrane in the acrosomal region are observed; (d) with missing acrosome, those spermatozoa in which the acrosomal region is not colored. In most cases the equatorial band of the sperm head may be stained light blue (Figure 3). For each sample, 100 spermatozoa were counted.

For the quantification of sperm abnormalities in fresh samples, smears stained with Coomassie blue were used and spermatozoa were classified as normal, with an abnormal head (pyriform or deformed, macro/microcephalic, bi/tricephalic), loose heads (normal and abnormal), abnormal midpiece (partial or total aplasia, bent with or without cytoplasmic droplet), and abnormal head/terminal piece (cut, bent with or without cytoplasmic gore) (Figure 3). A total of 100 spermatozoa were counted in each sample.

### 2.6. Statistical Analyses

Data were analyzed with SPSS version 11.5 (SPSS Inc., Chicago, IL, USA). The results are presented as mean ± standard error of the mean (SEM). Values of *p* < 0.05 were considered statistically significant. A split-plot ANOVA was used with a repeated measures factor, time, from fresh sample to post-thaw incubation, and a completely randomized fixed factor which in each study was the factor whose effect on the SMI and the percentage of sperm with intact acrosome was to be studied. The factors were the diluent, the speed of refrigeration (30 or 120 min), the storage system (straws or pellets), the temperature and timing of loading of the straws (before refrigeration at room temperature or after refrigeration at 5 °C), and the freezing system (in one or two levels on nitrogen vapors). For the study of the interactions between factors, split-plot ANOVA was used with time as a repeated measures factor and two inter-subject factors.

## 3. Results

### 3.1. Sperm Abnormalities in Epididymal Samples of Domestic Cat

The mean value for the percentage of normal spermatozoa (30.95 ± 1.98%) allows us to define the domestic cat sample used in this study as teratospermic (with <40% normal spermatozoa). About 25% of the samples were normospermic (with >40% normal spermatozoa; average 63.36 ± 2.74% normal spermatozoa) whereas the remaining samples were teratospermic (average 19.43 ± 0.89% normal sperm). Considerable differences were found between males (range of 5–90% normal spermatozoa).

The most frequent sperm abnormality was found in the principal and terminal pieces of the flagellum (36.7 ± 2.15% of spermatozoa), followed by abnormalities in the midpiece (27.7 ± 1.6% of spermatozoa). There was a low percentage of abnormal heads (<2%). Lastly, the proportion of spermatozoa with an intact acrosome exhibited a broad range (11–88%), with an average of 57.69 ± 1.40% (Table 2).

### 3.2. Effect of Cooling Rate: −0.125 °C/min vs. −0.5 °C/min

**Figure 4:**
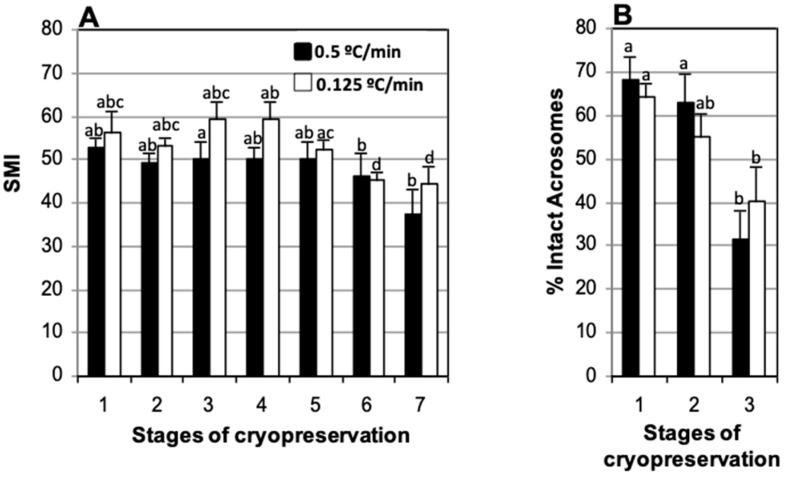
Effect of cooling rate (−0.5 and −0.125 °C/min) on motility and percentage of sperm with intact acrosomes of cat epididymal spermatozoa suspended in TEST 4% glycerol, stored in straws loaded at 5 °C, and frozen using two levels on nitrogen vapors. (**A**) Sperm Motility Index (SMI) and (**B**) percentage of sperm with intact acrosome. Timing of cryopreservation: (1) fresh, (2) after refrigeration, (3) after thawing, (4, 5, 6, 7) after incubation post-thaw for 90, 150, 210, and 270 min at 37 °C. For each cooling rate, different letters between bars indicate statistically significant differences (*p* < 0.05) in time. No significant differences were found between cooling rates (−0.5 vs. −0.125 °C/min) at any time during cryopreservation (*n* = 21).

To evaluate the effect of cooling speed on motility and acrosome integrity, samples extracted in TEST with 4% glycerol, refrigerated slowly (−0.125 °C/min) or rapidly (−0.5 °C/min), stored in straws loaded at 5 °C, and frozen using two levels over nitrogen vapors, were compared (Figure 4). In samples refrigerated for 30 min (−0.5 °C/min) or 120 min (−0.125 °C/min) the Sperm Motility Index (SMI) decreased gradually until 270 min post-thaw (37.5 ± 5.4 and 44.3 ± 4.0, respectively) at which time a significant decrease was observed when compared to samples assessed immediately after thawing (50.0 ± 4.3 and 59.2 ± 4.2, respectively) (Figure 4A). The percentage of spermatozoa with intact acrosomes after thawing decreased significantly in samples refrigerated for 30 min (−0.5 °C/min) and 120 min (−0.125 °C/min) (31.3 + 6.6% and 40.3 ± 7.8%, respectively) relative to fresh samples (68.1 ± 5.3% and 64.4 ± 3.1%, respectively). The decrease was more abrupt and marked in the samples refrigerated for 30 min and thus, in this case, we also found a significant drop in the percentage of intact acrosomes at thawing (31.3 ± 6.6%) relative to the values observed at the end of refrigeration (63.0 ± 6.6%) (Figure 4B). No significant differences in SMI or percentage of intact acrosomes were found between the two cooling rates (−0.5 and −0.125 °C/min) at any of the times considered (Figure 4).

### 3.3. Effect of Packaging Method: Straws vs. Pellets

**Figure 5:**
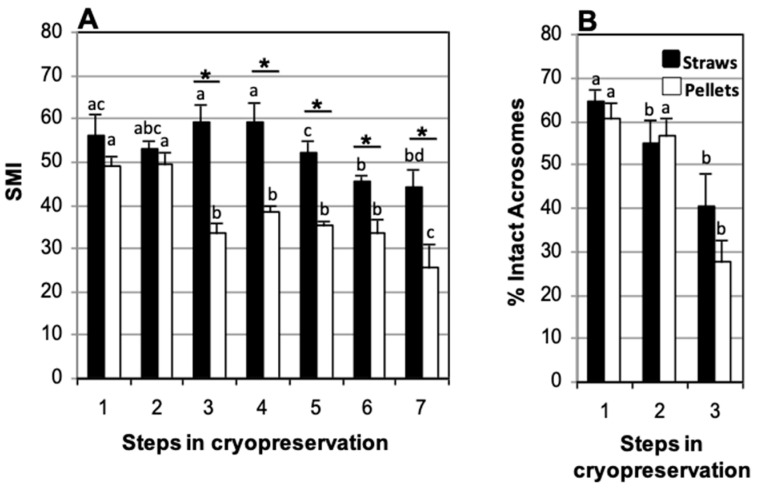
Comparison of storage vessels (straws or pellets) used for cat epididymal sperm cryopreserved in TEST with 4% glycerol and refrigerated for 120 min. (**A**) Sperm Motility Index (SMI) and (**B**) percentage of sperm with intact acrosome. Timing of cryopreservation: (1) fresh, (2) after refrigeration, (3) after thawing, (4, 5, 6, 7) at 90, 150, 210, and 270 min incubation post-thaw at 37 °C. Different letters between the bars indicate statistically significant differences (*p* < 0.05) over time (1–7), for each storage system (straws or pellets). * indicates statistically significant differences (*p* < 0.05) between pellets and straws (*n* = 19).

To study the effects of the storage vessel (pellets or straws) on motility and acrosome integrity, samples collected in TEST with 4% glycerol and refrigerated at 5 °C for 120 min were compared. No decrease in SMI upon thawing was found with regards to the fresh sperm suspension in samples frozen in straws (59.2 ± 4.2 and 56.3 ± 4.8, respectively). In contrast, a decrease was observed in sperm frozen in pellets (33.6 ± 2.4 SMI at thawing and 49.2 ± 2.0 SMI fresh) (Figure 5). A significant decrease in the percentage of spermatozoa with intact acrosome was observed between thawed and fresh samples, both in samples frozen in straws (40.3 ± 7.8% and 64.4 ± 3.1%, respectively) and in those frozen in pellets (27.6 ± 4.7% and 60.4 ± 3.7%, respectively) (Figure 5B). Statistically significant differences in SMI were observed between straws and pellets at thawing (59.2 ± 4.2 and 33.6 ± 2.4, respectively) that became more marked during post-thaw incubation (44.3 ± 4.0 and 25.8 ± 5.1, respectively, at 270 min of incubation) (Figure 5A). No statistically significant differences were found, however, in the percentage of spermatozoa with intact acrosome at thawing between samples frozen in straws or in pellets (40.3 ± 7.8% and 27.6 ± 4.7%, respectively) (Figure 5B).

### 3.4. Straw Loading Temperature: Room Temperature vs. 5 °C

**Figure 6:**
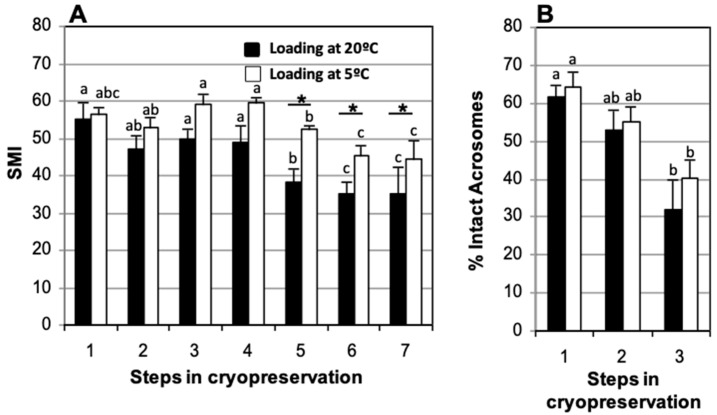
Comparison of straws loaded at different temperatures. Cat epididymal spermatozoa were cryopreserved in TEST 4% glycerol, after refrigeration for 120 min, with straws loaded at room temperature or at 5 °C. (**A**) Sperm Motility Index (SMI) and (**B**) percentage of spermatozoa with intact acrosome. Timing of cryopreservation: (1) fresh, (2) after refrigeration, (3) after thawing, (4, 5, 6, 7) at 90, 150, 210, and 270 min post-thaw at 37 °C. For each straw loading system (room temperature or 5 °C), different letters between bars indicate statistically significant differences (*p* < 0.05) over time. * indicates statistically significant differences (*p* < 0.05) between the two straw loading systems. Straws loaded at room temperature (*n* = 10), straws loaded at 5 °C (N = 10).

No significant decrease in SMI after thawing was found for the two methods analyzed (Figure 6A). Comparison of straws loaded at room temperature and at 5 °C showed significant decreases in the percentage of intact acrosome between fresh (61.6 ± 6.3% and 64.4 ± 3.1%, respectively) and post-thaw (31.8 ± 5.9% and 40.3 ± 7.8%, respectively) samples (Figure 6B). When straws were loaded at room temperature, a significant decrease in SMI relative to fresh samples (55.0 ± 4.4) was observed at 150 min post-thaw (38.2 ± 3.8) although no further decrease was observed at 210 min (35.0 ± 3.4) or 270 min (35.0 ± 7.2) of incubation (Figure 6A). No significant differences were found in SMI between samples loaded at room temperature or at 5 °C at the end of refrigeration (47.3 ± 3.4 and 53.0 ± 2.0, respectively), and after thawing (49.6 ± 3.0 and 59.2 ± 4.2, respectively) (Figure 6A). There were also no significant differences in the percentage of spermatozoa with intact acrosome at the end of refrigeration (52.7 ± 7.1% and 55.1 ± 5.3%, respectively) or after thawing (31.8 ± 6.0% and 40.3 ± 7.8%, respectively) (Figure 6B).

### 3.5. Effect of Freezing at One or Two Levels over Nitrogen Vapors

**Figure 7:**
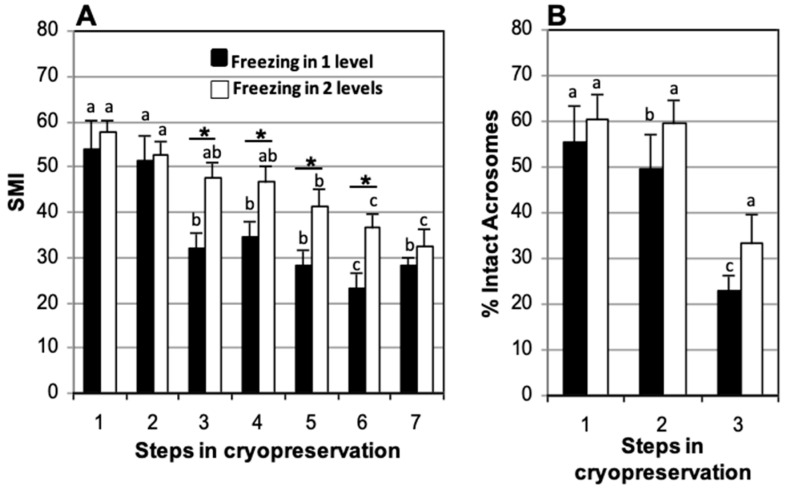
Comparison of one- or two-level freezing in nitrogen vapors of domestic cat sperm cryopreserved in TEST 4% glycerol, refrigerated for 120 min, and stored in straws loaded at 5 °C. (**A**) Sperm Motility Index (SMI) and (**B**) percentage of sperm with intact acrosome. Timing of cryopreservation: (1) fresh, (2) after refrigeration, (3) after thawing, (4, 5, 6, 7) at 90, 150, 210, and 270 min incubation post-thaw at 37 °C. For each freezing method (one level or two levels), different letters between bars indicate statistically significant differences (*p* < 0.05) in time. * indicates statistically significant differences (*p* < 0.05) between the two freezing methods (one or two levels) (*n* = 15).

In samples frozen using one level over nitrogen vapor, significant differences in SMI appeared after thawing (32.2 ± 3.4) when compared to fresh samples (54.1 ± 6.3), which remained during post-thaw incubation for 150 min (28.1 ± 3.6) and 210 min (23.2 ± 3.2). In the samples frozen using two levels, there were no significant differences in SMI in relation to fresh samples (57.7 ± 2.5) until after 150 min post-thaw (41.4 ± 3.5) which increased after 210 min of incubation (36.4 ± 3.3) (Figure 7A). Regardless of whether the straws were frozen in one or two levels, significant differences were found in the percentage of spermatozoa with intact acrosome after thawing (22.9 ± 3.2 and 33.3 ± 6.2%, respectively) with respect to the fresh samples (55.6 ± 7.9 and 60.5 ± 5.3%, respectively) and samples assessed after refrigeration (49.6 ± 7.7 and 59.8 ± 5.0%, respectively) (Figure 7B).

The freezing system produced significant differences in SMI at thawing in favor of samples frozen at two levels relative to those frozen at one level (47.5 ± 3.4 and 32.2 ± 3.4, respectively), which were maintained during incubations post-thaw (36.4 ± 3.3 and 23.2 ± 3.2, respectively, at 210 min of incubation) (Figure 7A). No significant differences were found in the percentage of spermatozoa with intact acrosome at thawing between samples frozen at one or two levels (22.9 ± 3.2% and 33.3 ± 6.2%, respectively) (Figure 7B).

### 3.6. Effect of Interactions Between Factors in the Cryopreservation Process

The possible interactions (two by two) between some of the factors analyzed individually in the previous sections were studied. The possible interactions between cooling rate (−0.5 or −0.125 °C/min) and the storage vessel (straws or pellets) and loading temperature of the straws (room temperature or 5 °C) were examined. None of the interactions involving the cooling rate were found to be significant (Table 3 and Table 4).

## 4. Discussion

The results of this study provide insight on the effects of four key factors important for the cryopreservation of epididymal spermatozoa from domestic cats, which serves as the main experimental model for endangered felids. The analysis of the effect of refrigeration rate on the motility and acrosomal integrity of cryopreserved cat epididymal spermatozoa indicated that the rate of refrigeration appeared to have a greater impact on acrosomal integrity than on motility. When two refrigeration rates (−0.5 °C/min and −0.125 °C/min) were tested, SMI values remained stable during cryopreservation but declined after 270 min of incubation, with a more abrupt decrease in the shorter refrigeration time. Additionally, assessments of acrosome integrity showed that, after thawing, spermatozoa subjected to slow refrigeration (120 min) had a higher, though not statistically significant, percentage of intact acrosomes compared to those subjected to fast refrigeration (30 min).

Our results align with previous studies [47] which showed greater resilience in motility but increased sensitivity of acrosome integrity to cold shock damage [48]. In the aforementioned study [47], it was observed that ultra-fast cooling at 14 °C/min and fast cooling at 4 °C/min resulted in significant differences upon returning to the initial temperature compared to the fresh sample, whereas no such differences were observed at a slower cooling rate of −0.5 °C/min [47]. Thus, faster cooling rates are more detrimental than slower ones, particularly between 20 °C and 0 °C, which is when the most profound phase changes occur in the lipids of the sperm membrane [49]. However, other studies on domestic cat epididymal spermatozoa [34] found no significant differences in the percentage of intact acrosomes upon returning to room temperature when comparing faster and slower cooling rates. This lack of difference is likely due to the minimal variation between the rates tested (−3 °C/min vs. −4 °C/min).

Studies on wild felids have shown that while motility was somewhat affected by the use of faster or slower cooling rates (−0.7 °C/min and −0.16 °C/min), acrosomal integrity was more significantly impacted (ocelot, *Leopardus pardalis*: [50]). Surprisingly, in the ocelot, the best acrosome integrity results at thawing were obtained using a faster cooling rate (−0.7 °C/min) rather than a slower one (−0.16 °C/min), which contrasts with findings in the domestic cat. In the case of the oncilla (*Leopardus tigrinus*), although the reduction in the proportion of spermatozoa with intact acrosomes at thawing was not significantly lower than that in the fresh sample, the trend aligned with that observed in the domestic cat. A higher proportion of spermatozoa with intact acrosomes was maintained at thawing when a slower cooling rate was used [50]. From these results, it can be concluded that, although the general trend suggests that faster cooling rates are more detrimental than slower ones, as described by Watson [49], and confirmed in studies with domestic cats, the opposite effect may occur in certain species, at least for cooling rates between −0.16 °C/min and −0.7 °C/min.

In the present study, significant differences were found in the SMI at thawing due to the sperm packaging system, with straws being better than pellets, in agreement with Pope et al. [33] who observed a decrease of 76% in motility and 66% of normal acrosomes in semen frozen in pellets. These authors concluded that straws (decrease of 43% in motility and 30% in normal acrosomes) are better for cat sperm cryopreservation when compared to pellets. The importance of the proportion of normal and acrosome-intact spermatozoa as indicators of survival after thawing was confirmed, and a good relationship was also observed between the proportion of motile spermatozoa before and after freezing.

When comparing straws loaded at room temperature before refrigeration with those loaded at 5 °C after refrigeration, a lower (but not significant) SMI and a lower percentage of sperm with intact acrosomes were found in the former (loading at room temperature). During post-thaw incubation (from 1.5 h to 4.5 h), significantly lower SMI values were observed in samples loaded at room temperature (before refrigeration). These results may be due to the fact that when straws are loaded before refrigeration, the volume of sperm suspension subjected to the temperature drop (in 50 µL/straw) is smaller than when straws are loaded after refrigeration, as the entire sample (~300 µL) is cooled in a microtube to 5 °C. Therefore, the temperature drop is likely faster in the former, subjecting the spermatozoa to a more acute thermal stress. Despite these results, it should be noted that under field conditions it is generally not possible to load the straws at 5 °C, as the available refrigerators do not offer the necessary conditions for handling semen, or sometimes no refrigerator is available. For these reasons, it will be necessary to anticipate and accept the slight decrease in semen sample quality that may result from loading straws at room temperature. This may help compensate for any quality loss that may occur if epididymides or samples are transported to the laboratory for an extended period of time before sperm collection and processing [51].

Finally, the effect of the method for freezing straws in nitrogen vapors was examined. Straws were either frozen at one level (5 cm above the surface of the liquid nitrogen for 10 min) or at two levels (7.5 cm and 2.5 cm above the surface of the liquid nitrogen, for 1 min each). Freezing using the two-level system resulted in significantly better outcomes for motility and acrosome integrity than the one-level freezing, as observed both at thawing and during subsequent incubation. These results are consistent with those obtained in other studies in domestic cat ejaculated sperm, where slower freezing rates (from −3.85 to −43 °C/min, between 5 °C and −40 °C) resulted in higher percentages of motile and acrosome-intact sperm at thawing than faster rates [35].

## 5. Conclusions

When performing cryopreservation of domestic cat epididymal spermatozoa, we found that (1) the use of fast (−0.5 °C/min) or slow (−0.125 °C/min) refrigeration rates does not produce significant differences in SMI or in the percentage of intact acrosomes, (2) storing sperm in straws results in significantly higher post-thaw SMI values compared to storing in pellets, (3) loading straws at 5 °C leads to better SMI values, (4) using a two-level straw freezing system over nitrogen vapors provides significantly higher SMI values at thawing than samples frozen at a single level, and (5) there is no significant interaction between SMI and acrosomal integrity based on the temperature at which the straws are loaded. These results indicate that by selecting adequate parameters, the success of cryopreservation of epididymal cat sperm can be improved. Furthermore, the use of some simpler procedures may still provide adequate means of cryopreservation of felid sperm under field conditions.

## Figures and Tables

**Table 1:** Stages of cryopreservation process in which samples were taken for sperm evaluation.

Stage	Status of Sample
1	Fresh from the epididymis
2	After refrigeration, before freezing
3	Soon after thawing
4	90 min incubation post-thawing
5	150 min incubation post-thawing
6	210 min incubation post-thawing
7	270 min incubation post-thawing

**Table 2:** Abnormalities of sperm morphology found in domestic cat in fresh samples.

Parameter	Mean ± SEM
Total number of spermatozoa (×10^6^)	28.15 ± 3.23
Normal spermatozoa (%)	30.95 ± 1.98
Head abnormalities (%)	1.41 ± 0.21
Medium piece abnormalities (%)	27.72 ± 1.63
Principal and terminal piece abnormalities (%)	36.70 ± 2.15
Coiled tail (%)	4.62 ± 0.77
Cytoplasmic droplet (%)	27.89 ± 2.38
Intact acrosomes (%)	57.69 ± 1.40

**Table 3:** Interactions between cryopreservation factors and time on Sperm Motility Index (SMI).

			Double Interactions		Triple Interaction	
SMI			*F*	*p*	*F*	*p*
1. Time	2. Refrigeration	3. Storage system	(1–2) 0.44	0.81	(1.2.4) 2.58	0.07
			(1–4) 1.24	0.34		
			(2–4) 0.09	0.77		
		4. Straw’s loading	(1–2) 1.83	0.15	(1.2.5) 0.57	0.72
			(1–5) 0.67	0.65		
			(2–5) 1.81	0.19		

Numbers between brackets point out factors involved in the interactions, followed by split-plot ANOVA statistics (*F* and *p*).

**Table 4:** Interactions between cryopreservation factors and time on proportion of spermatozoa with intact acrosome.

			Double Interactions		Triple Interaction	
% Intact Acrosomes			*F*	*p*	*F*	*p*
1. Time	2. Refrigeration	3. Storage system	(1–2) 0.70	0.51	(1.2.4) 0.18	0.84
			(1–4) 0.43	0.06		
			(2–4) 0.26	0.62		
		4. Straw’s loading	(1–2) 0.32	0.63	(1.2.5) 0.49	0.62
			(1–5) 0.53	0.60		
			(2–5) 0.02	0.88		

Numbers between brackets point out factors involved in the interactions, followed by split-plot ANOVA statistics (*F* and *p*).

## Data Availability

The original contributions presented in this study are included in the article. Further inquiries can be directed to the corresponding author(s).
